# A Narrative Review on Unravelling Bacterial‐Mediated Carcinogenesis and Possible Alternative Treatment Strategies

**DOI:** 10.1155/bmri/6359088

**Published:** 2026-04-07

**Authors:** Md Sohel, Suraiya Aktar, Sanzida Khatun, Sherif Hamidu, Nishat Ulfat Nity, Sandeep Kumar, Md Sakhawat Hossain, Md. Rifat Sarker, Snygdha Rani Das, Badhan Rani Dey, Ali Mohamod Wasaf Hasan, Khairul Islam, Farhadul Islam, Abdullah Al Mamun

**Affiliations:** ^1^ Biochemistry & Molecular Biology, Mawlana Bhashani Science & Technology University, Tangail, Bangladesh, mbstu.ac.bd; ^2^ Biochemistry and Molecular Biology, Primeasia University, Dhaka, Bangladesh, primeasia.edu.bd; ^3^ Biochemistry and Molecular Biology, Rajshahi University, Rajshahi, Bangladesh, ru.ac.bd; ^4^ Department of Clinical Pathology, Noguchi Memorial Institute for Medical Research, University of Ghana, Accra City, Ghana, ug.edu.gh; ^5^ Department of Genetics, University of Alabama at Birmingham, Birmingham, Alabama, USA, uab.edu; ^6^ Biotechnology and Genetic Engineering, Faculty of life science, Mawlana Bhashani Science and Technology University, Tangail, Bangladesh, mbstu.ac.bd; ^7^ Department of Pharmacy, Mawlana Bhashani Science & Technology University, Tangail, Bangladesh, mbstu.ac.bd

**Keywords:** bacterial carcinogenesis, DNA damage, inflammation, microbiome–cancer axis, nanotechnology, oncogenic bacteria, phytochemicals, tumorigenesis

## Abstract

The potential roles of chemical, physical, and viral factors in cancer development are well documented. Similarly, bacterial carcinogenesis has been reported previously, though not extensively. Therefore, we aimed to provide comprehensive, mechanistic evidence on the pathogenesis of bacteria‐induced carcinogenesis and possible treatments to halt cancer progression. Infections by bacteria, including *Salmonella typhi*, *Fusobacterium* spp., *Chlamydia pneumoniae*, *Staphylococcus aureus*, *Helicobacter pylori*, and *Mycobacterium tuberculosis*, have been reported as the most common carcinogenic bacteria in humans. These bacteria can produce toxins and carcinogenic metabolites those promote the development of cancer in a variety of ways, including by changing the dynamics of the cell cycle, triggering signaling pathways in the cell, such as NF‐*κ*B, MAPK, PI3K‐PKB/Akt, and JAK/STAT, and activating anti‐apoptosis activities by increasing Bcl‐2 and decreasing BAX, and caspases expression along with suppressing p53 and pRb tumor suppressor proteins. Moreover, inflammatory cytokines such as tumor necrosis factor‐*α* (TNF‐*α*), interferon‐gamma (INF‐*γ*), interleukin‐1*β* (IL‐1*β*), IL‐4, IL‐6, IL‐10, IL‐1, IL‐17, IL‐23, and other inflammatory cytokines are a few of the factors that promote chronic inflammation and initiate carcinogenesis. In addition, bacterial infection can generate free radicals that induce DNA damage, thereby promoting carcinogenesis. Following these mechanisms, bacteria can cause a wide range of cancers, such as breast, colon, pancreas, stomach, lung, gallbladder, and oral carcinoma. Fortunately, supplementation with active natural phytochemicals and nano‐based strategies may counteract bacterial infection‐induced carcinogenesis by regulating several cellular proteins, including those that control the cell cycle, induce apoptosis, promote metastasis, interact with growth factor receptors and tyrosine kinases, and function as antioxidants. Therefore, this narrative review aims to provide a consolidated mechanistic overview of bacterial infection‐induced carcinogenesis and to highlight emerging phytochemical and nanotechnology strategies as potential therapeutic approaches. Additionally, phytochemical‐based interventions and nanotechnology strategies are discussed as potential alternative therapeutic approaches to counteract bacteria‐induced carcinogenesis.

## 1. Introduction

Cancer is the second leading cause of death globally, with 10 million cancer‐related deaths and 20.1 million new cases reported [1]. According to global cancer epidemiological data, lung, prostate, colorectal, and stomach cancers represent the most prevalent malignancies in men, while breast, colorectal, lung, and cervical cancers are the leading cancer types in women [2]. Human cancer is primarily caused by environmental, genetic, epigenetic, and dietary factors [3]. In addition to this, commensal microbes, which generally support health, can lead to diseases such as cancer when imbalanced [4]. The normal functioning of our digestive systems and the prevention of pathogen colonization are greatly aided by the commensal bacteria in our bodies. But the imbalance among these microbes, known as bacterial dysbiosis, can alter a number of host cellular signaling pathways, resulting in the emergence of a wide range of illnesses, including cancer and antibiotic resistance, and ultimately leading to death [5]. The intricate relationships between microorganisms and their host cells make it difficult to define and quantify the contribution of the microbiota to cancer progression [6]. Although tumors associated with oncogenic viruses have been studied extensively, in‐depth research into the role of bacteria in oncogenesis is lacking. There are approximately 3.9 × 10 [7] bacterial cells in an average human aged 20–30, with 70 kg of body weight and a height of 170 cm, which is almost equal to the host cell counts in the human body [8]. Thus, it is thought that this high bacterial load may be associated with the pathogenesis. Therefore, studies about the association between bacterial infection and carcinogenesis revealed that an etiologic association between the onset of cancer and bacterial infection is prevalent in many patients with cancer [9]. For example, beginning in 1868, two patients with sarcoma were identified as infected with *Streptococcus*, and infection with *S. pyogenes* was detected in a patient with neck cancer [10]. There have been reports of a correlation between the occurrence of certain malignancies and diseases caused by specific bacterial species, such as *S. aureus* infection with breast cancer (BC) [11], *H. pylori* infection with gastric cancer (GC) [12], *Fusobacterium* spp. and colibactin expressing *Escherichia coli* infection with colorectal cancer (CRC) [7, 13], *Mycobacterium tuberculosis* infection with lung cancer (LC) [14], *Porphyromonas*, *Prevotella*, and *Fusobacterium* spp infection with oral cancer (OC) [15, 16] and so on. Collectively, the above evidence indicates that bacterial infections are linked to the development of cancer. Nevertheless, there is no consensus on the molecular pathways by which bacterial infections contribute to the development of different cancer types. The molecular pathways linking bacterial infections to cancer remain unclear, requiring further investigation to identify precise mechanisms. Understanding the mechanisms by which bacterial infections contribute to carcinogenesis is crucial for developing effective preventive and therapeutic strategies. Although antibiotics are the first‐line treatment for bacterial infections, their full benefits for patients with cancer are not yet fully understood. Additionally, supportive care, hospitalization, and surgery may be just options for bacterial‐mediated cancer treatments. However, discovering the unique treatment approaches that are more effective for bacterial‐mediated carcinogenesis. So, exploring novel therapeutic strategies, particularly plant‐derived phytochemicals and nano‐based strategies, may be possible ways to target bacterial‐mediated carcinogenesis [17, 18]. Therefore, this review aims to summarize mechanistic insight into bacterial infection‐induced carcinogenesis. Also, outlined potential phytochemicals and nanotechnology‐based interventions with protective effects against bacterial infection‐induced carcinogenesis across various cancers.

## 2. Methodology

This work is a narrative review synthesizing current evidence on bacterial‐mediated carcinogenesis and emerging therapeutic strategies. A comprehensive literature search was conducted in PubMed, Scopus, Web of Science, and Google Scholar for studies published between 2000 and 2025. Searching keywords including bacterial carcinogenesis, oncogenic bacteria, microbiota and cancer, phytochemicals, nanoparticles, bacterial infection cancer mechanism, and microbiome‐induced cancer.

### 2.1. Eligibility Criteria

For this narrative review, we focused on peer‐reviewed scientific publications, including original research articles, systematic reviews, meta‐analyses, in vitro and in vivo experimental studies, and clinical reports. Studies were considered if they discussed mechanisms of bacteria‐induced carcinogenesis or explored therapeutic interventions such as phytochemicals or nanotechnology and were available in English. Publications unrelated to cancer biology, general microbiology without oncologic relevance, non‐scientific commentaries, and opinion pieces were not considered.

### 2.2. Synthetic Method

Given the narrative nature of this review, findings from eligible studies were synthesized qualitatively. Mechanistic insights were grouped into major themes, including inflammatory pathways, DNA damage, regulation of apoptosis, and oncogenic signaling. Therapeutic evidence was summarized based on reported molecular targets and anticancer outcomes.

## 3. Result and Discussion

### 3.1. A General Mechanistic Overview of Bacterial Infection‐Induced Carcinogenesis

Bacterial infection can induce carcinogenesis in several ways, such as producing carcinogenic metabolites and toxins, causing chronic inflammation, targeting tumor‐specific intracellular signaling, etc. For example, the peptidoglycan (PGN) produced by *S. aureus* in bacteria [19] and *H. pylori* [20–22] are linked to the pathophysiology of gastric and BCs, respectively. Furthermore, bacterial secretion of estrogen, lipopolysaccharides (LPS), N‐nitrosamines etc., could act as carcinogenic metabolites that produce and cause breast [23, 24], and pancreatic [25]. These metabolites can interact with a number of Toll‐like receptors (TLRs), which are essential for controlling the innate immune system, which in turn modulates the signaling pathways involved in the initiation and progression of cancer. Bacterial infections may lead to the generation of toxins that cause DNA damage and promote tumorigenesis (comparative analysis of cytolethal distending toxin (cdt) genes among *Campylobacter jejuni*, *C. coli*, and *C. fetus* strains). For example, *C. jejuni* produces cyto‐lethal distending toxins associated with the pathogenesis of CRCs [15]. Moreover, infection with *H. pylori* is linked with GC [26] and LC [27] by producing virulence factors, such as the cytotoxin‐associated gene (CagA), which is composed of VacA and CagPAI. These bacterial toxins change cellular growth and cause the cell cycle to become dysregulated [28], thereby inducing cancer pathogenesis. Also, pathogenic bacteria attribute cancer pathogenesis by targeting tumor‐specific intrinsic intracellular signaling pathways that can enhance pathogen survival [29]. The control of these signaling factors is crucial in the growth or prevention of tumors. For example, *β*‐catenin is a common signaling pathway targeted by *Fusobacterium nucleatum* and is associated with the pathogenesis of colorectal [30, 31], and oral carcinoma [32]. Moreover, bacterial infections induce cell proliferation through activating JAK/STAT [33], NF‐*κ*B, and MAPK cascade, including Ras, RAF, E‐MEK, ERK1/2, and PI3K‐PKB/Akt pathways [34, 35]. Furthermore, intracellular pathogen buildup brought on by bacterial infections may decrease apoptosis, mainly by altering the expression of the proteins Bcl2, BAX, and caspases or by deactivating the retinoblastoma protein, p53, and pRb [36, 37]. Moreover, chronic inflammation is an important factor in the development of cancer, accounting for 25% of all cancer cases in humans [38]. The bacterial infection triggers chronic inflammation at the infection site, promoting the secretion of various inflammatory mediators. For instance, interleukin‐1*β* (IL‐1*β*) and TNF‐*α* are well documented for their roles in amplifying immune responses and facilitating cancer progression, as shown in studies involving compounds such as formononetin and cucurbitacin B [39, 40]. IL‐6 and IL‐10, key regulators of pro‐ and anti‐inflammatory balance, have been linked to modulation of the STAT3 signaling pathway in several cancers [41, 42]. Additionally, cytokines such as IL‐4, IL‐17, and IL‐23 are implicated in the tumor microenvironment’s immune regulation, influencing both tumor suppression and immune evasion. IFN‐*γ* has also been highlighted for its dual role in promoting antitumor immunity and contributing to chronic inflammation, as seen in genistein‐ and resveratrol‐based studies [40]. These pro‐inflammatory cytokines activate oncogenic signaling pathways, including STAT3, Smad3, and NF‐*κβ*, triggering uncontrolled cell proliferation. Moreover, by activating enzymes like nitric oxide synthase, superoxide dismutase (SOD), and NADPH (nicotinamide adenine dinucleotide phosphate) oxidase, these immunological chemicals also increase reactive oxygen species (ROS) and nitrogen oxide species (NOS). Bacterial pathogens stimulate enzymes such as NADPH oxidase and inducible nitric oxide synthases (iNOS), leading to excessive production of ROS and reactive nitrogen species (RNS). These reactive molecules attack DNA, causing strand breaks, oxidative base modifications (e.g., 8‐oxo‐dG), and chromosomal instability that promote carcinogenesis. These free radicals and derived metabolic products damage DNA and mediate uncontrolled cell division by escaping the cell cycle checkpoint and repair system, leading to the progression of cancer development. Altogether, pathogenic bacterial infections can accelerate DNA damage and induce cancer development.

### 3.2. Bacteria as Causative Agents of Various Cancers

Cancer is neither contagious nor a disease that can be spread. However, bacterial infections increase the risk of developing cancer. Previously, bacterial infection was not considered a significant route of cancer. However, advanced research exhibited the association, observed in a significant number of patients with cancer, between bacterial infections and the pathogenesis of cancer.

#### 3.2.1. BC

BC is the top carcinoma among women and is a leading cause of cancer‐related death worldwide [43]. According to Global Cancer Statistics 2020, around 11.7% and 6.9% of new cases and deaths are associated with BC, respectively [1]. A wide range of risk factors, including hormone replacement therapy, familial history, obesity, and personal habits, are associated with BC pathogenesis [44, 45]. In addition, accumulating information suggested that bacterial infection could initiate BC [46]. For example, Eslami et al. reported that bacterial factors such as gut composition, breast tissue, and milk microbiome, estrogen generation, and other metabolites could induce the development and progression of bacterial infection‐mediated BC pathogenesis [46]. These include bacterial components such as PGN, LPS, short‐chain fatty acids, *β*‐glucuronidase, and estrogen‐modulating microbial enzymes. It was reported that bacterial infections (e.g., infection by *Proteobacteria*, *Firmuculates*, *Actinobacteria*, *Methylobacterium*, *Bacteroidetes*, *Protobacteria*, *Firmicutes*, *Bacillus*, *Actinobacter*, *Bifidobacterium*, *Blautia*, *Firmicultes*, *Bacteriodetes*, *Clostridium*, *Cocciids Cluster*, *Bluster* spp.) were associated with the pathogenesis of BC.

**Figure 1:**
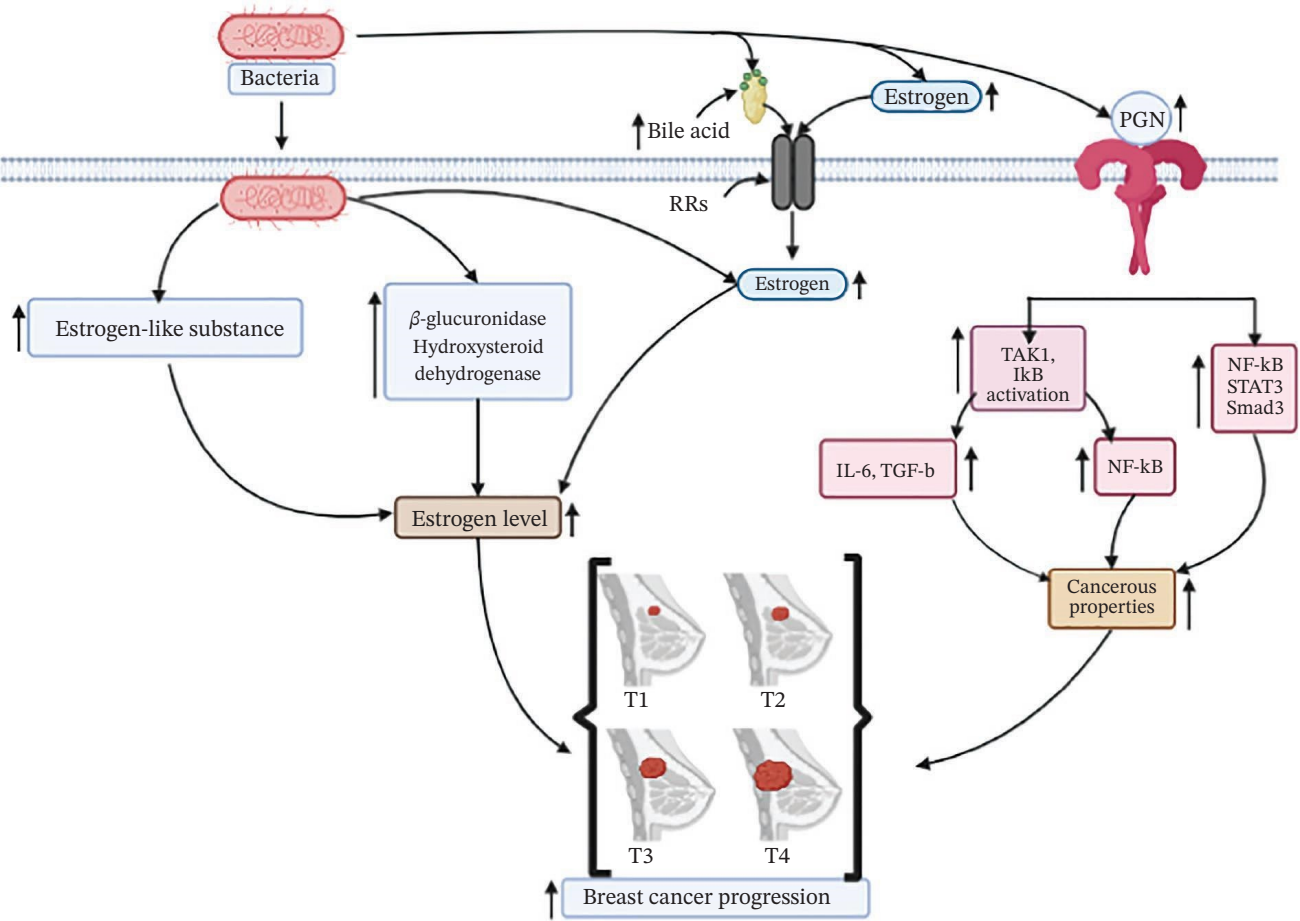
Schematic representation of breast carcinogenesis by bacterial infection. Bacteria secreted metabolites PGN phosphorylate TAK1 and IkB in the TLR2‐NF‐*κ*B pathway, which induce secretion of IL‐6 and TGF‐b and activate NF‐*κ*B, STAT3 and Smad3 in breast cancer. Bile acid secreted by bacteria‐reactivated estrogen and enter the breast cell, increasing cell proliferation, and some of them produce estrogen or estrogen‐like substances resulting in cancer initiation and progression.

It is still unknown how precisely microorganisms cause BC to progress. However, the role of gut microbiome in cancer development has been reported. Although the precise microbial cause of BC remains unresolved, accumulating evidence supports a gut–breast axis whereby gut dysbiosis alters systemic estrogen metabolism, immune regulation, and inflammatory signaling that influence breast tissue homeostasis. Proteoglycan (PGN), short‐chain fatty acid, reactivated estrogen, and bile acid are examples of bioactive metabolites that the gut microbiota may release and which have the potential to form cancerous bonds [46, 47]. For example, *S. aureus* secretes PGN, called PGN‐SA, which accelerates TLR‐2, promoting carcinogenic properties, including the invasiveness and adhesiveness of cancer cells [48]. PGN‐SA also phosphorylates TAK1 and IkB in the TLR2‐NF‐*κ*B pathway and induces secretion of IL‐6 and TGF‐b in BC (MDA‐MB‐231) cells [48]. Furthermore, PGN‐SA causes the activation of oncogenic signals such as NF‐*κ*B, STAT3, and Smad3, which can play an important role in BC formation [48]. Bile acid, another essential metabolite, can influence the growth and steroid receptor function of BC (MCF7) cells [49], and reactivated estrogen enters the breast cell, increasing cell proliferation. Moreover, bacteria may create estrogen or estrogen‐like compounds (such as steroid hormones) during metabolism, which increases the risk of estrogen‐dependent female BC, especially among women who have gone through menopause. Furthermore, gut microorganisms have the ability to encode enzymes that deconjugate estrogens, such as hydroxysteroid dehydrogenase and *β*‐glucuronidase. These enzymes can increase estrogen metabolism, improve its excretion, and release it into hepatic circulation in an active form, which may cause hormonal imbalance and accelerate the onset of BC. The increased estrogen or estrogen‐like substances, cellular proliferation through either receptor or hormonal mediated, are activated, resulting in cancer initiation and progression. A graphic overview of bacteria‐induced BC is summarized in Figure 1.

#### 3.2.2. CRC

Another prevalent form of cancer is CRC, which will account for 1.9 million new cases and 0.9 million deaths globally in 2020[1]. It has the third highest incidence rates and second highest cancer‐related mortality worldwide [50]. Risk factors associated with CRC include but are not limited to Westernized ways of life and diet, such as tobacco, alcohol consumption, and high red meat intake [51–54]. In addition, microbiota, especially gut microbiota, play crucial roles in the pathogenesis of colorectal carcinoma [55, 56]. A number of bacterial species, including *Streptococcus bovis* [57], *Helicobacter pylori* [58], *Bacteroides fragilis* [59], *F. nucleatum* [60], *Clostridium septicum*, *Klebsella pneumonae* [61], colibactin‐expressing pks + *E. coli* [62], are involved in the pathogenesis of colorectal carcinogenesis.

**Figure 2:**
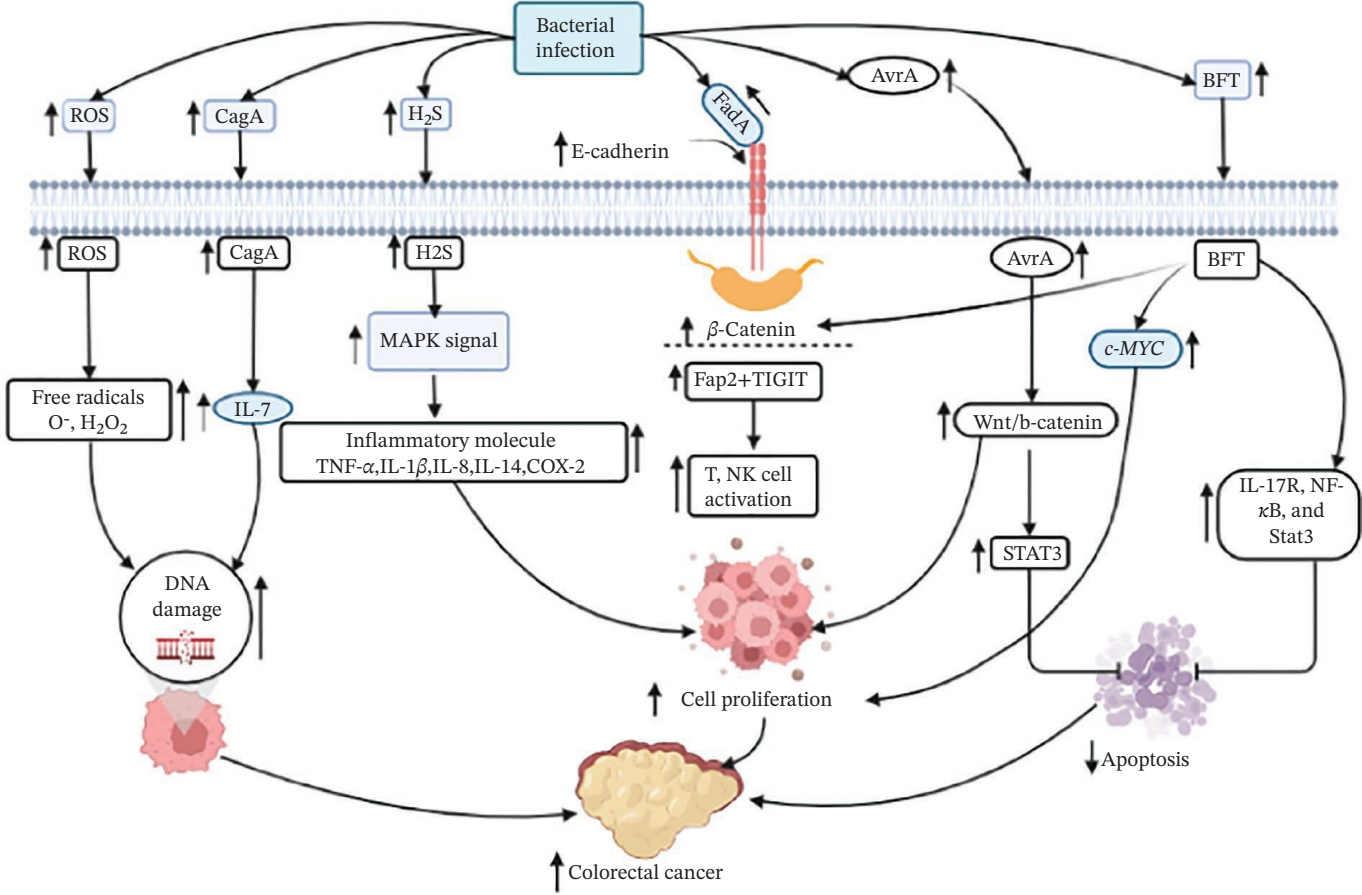
The bacterial infection induced CRC carcinogenesis. There are six mechanisms involved in colon carcinogenesis, which are regulated by bacteria induced secretion of numerous molecules. These secreted molecules are effectively pass the colon cell wall of host and start carcinogenesis by damaging DNA, activating inflammatory cytokines and some immune cells, preventing apoptosis and inducing cell proliferation.

Recent studies showed the mechanism by which intestinal microbiota induced the development of CRC [62]. Intestinal microbes, especially bacteria or their virulence factors such as genotoxins, bacterial metabolites, and oxidative stress generated by bacterial infection, share several common pathways, which in turn promote CRC genesis [63–65]. For instance, *F. nucleatum* causes CRC via its distinct adhesin A (FadA), which binds to E‐cadherin and activates the *β*‐catenin signaling pathway, promoting tumor growth and subsequent inflammation [66]. Fap2, the surface adhesin of *F. nucleatum,* binds to the immunological inhibitory receptor T‐cell immunoglobulin and ITIM domain, therefore inhibiting T‐cell activation and natural killer cell‐mediated cytotoxicity [67]. A group of sulfidogenic bacteria can produce hydrogen sulfide (H_2_S), followed by infection, which can cause DNA damage, making the chromosome unstable and accumulating a high mutation rate in cells [68]. In addition, H_2_S could activate the MAPK pathway, thereby activating the pro‐survival pathway leading to CRC development [69]. *S. bovis* can increase the production of NF‐*κβ*, IL‐14, IL‐8, COX‐2, Scyb1, Ptgs2, IL‐1*β*, TNF, and Ccl2 followed by infection [70–72]. These chemicals have been linked to a reduction in the rate of apoptosis, angiogenesis, and enhanced cell proliferation, all of which contribute to the development of cancer. Bacteroides fragilis toxin (BFT) generated in ApcMin mice with *S. bovis* infection triggers a pro‐carcinogenic, multi‐step inflammatory cascade followed by IL‐17R, NF‐*κ*B, and Stat3 signaling activation in colonic epithelial cells, resulting in myeloid‐cell‐dependent distal colon tumorigenesis via inhibition of apoptosis [73, 74]. Moreover, BFT cleaves epithelial cadherin (E‐Cadherin), which raises the amount of *β*‐catenin accumulation in the nucleus and causes the cellular‐myelocytomatosis (c‐MYC) oncogene to be overexpressed, which in turn encourages persistent cell proliferation [75]. Another mouse model study by Dejea et al. reported that mouse colon colonized with *E. coli* (expressing colibactin), and enterotoxigenic *B. fragilis* showed increased interleukin‐17 in the colon and DNA damage in colonic epithelium [76]. These bacterial infection‐mediated alterations are associated with faster tumor onset and significantly increased animal mortality compared with the control. Furthermore, *E. coli* produces a bacterial adhesion protein called intimin, which could promote carcinogenesis by downregulating the DNA mismatch repair gene [77]. Also, *H. pylori* infection can generate pathogenic factors such as CagA, which can activate oncoprotein through gastric Src family kinase, resulting in SHP_2_‐activated transient signal for cell overgrowth, motility, and other properties to promote carcinogenesis [78, 79]. Moreover, these bacteria can produce oxidative ROS, pro‐inflammatory cytokines, and COX‐2 and superoxide radicals, promoting colonic carcinogenesis [80]. *Enterococcus faecalis* play a critical role in CRC pathogenesis via the production of free radicals like superoxide (o‐), and hydrogen peroxides (H_2_O_2_), which can damage colonic epithelial cells’ DNA via a bystander effect [81]. Also, infection of these bacteria causes chronic inflammation through activating IL‐10 pathways and associated with genome rearrangements and chromosomal instability (18), including aneuploidy and tetra‐ploidy in colonic epithelial cells. When gram‐negative bacteria, including *Salmonella* species, invade colonic tumor cells, they activate the Wnt/*β*‐catenin and STAT3 signaling pathways, which raises the risk of cancer growth [82]. A cytolethal distending genotoxin produced by *Campylobacter* jejuni damages double strands of DNA, causing a rise in CRC. Also, *P. anaerobius* stimulates TLR2/TLR4 and may increase intracellular ROS levels, which would encourage biosynthesis and cell division [83]. Figure 2 provides a summary of the graphic representation of bacteria‐induced CRC.

#### 3.2.3. Pancreatic Cancer (PC)

PC is one of the deadliest cancers, and the 5‐year survival rate is only 11% in patients with this cancer [84]. Well‐known risk factors for PC are tobacco smoking [85], chronic pancreatitis, type 2 diabetes, inflammation, ABO type gene, etc. [86–88]. Furthermore, bacterial infection and the etiology of PC were found to be positively correlated in preclinical and clinical investigations. Oncogenic bacteria such as *H. pylori* [87], *Porphyromonas gingivalis* [87]*, Porphyromonas, actinomycetes*, and *Neisseria* [89] may have an impact on the development and recurrence of PC. Bacterial metabolites activate several pro‐inflammatory mediators and signaling pathways, stimulating PC pathogenesis. For example, LPS and lipoteichoic acid (LTA) are components of bacteria that can interact with the TLRs signaling pathway and CD14, thereby triggering the NF‐*Ρ*B and MAPK pathways, which in turn trigger the synthesis of cytokines and further recruitment of pro‐inflammatory elements, resulting in initiation and development of carcinogenesis in the pancreas [90, 91]. In addition, LPS‐TLR signaling can activate STAT3 and promote Kirsten rat sarcoma viral oncogene (KRAS) activation, constituting cellular proliferation [92–94].

**Figure 3:**
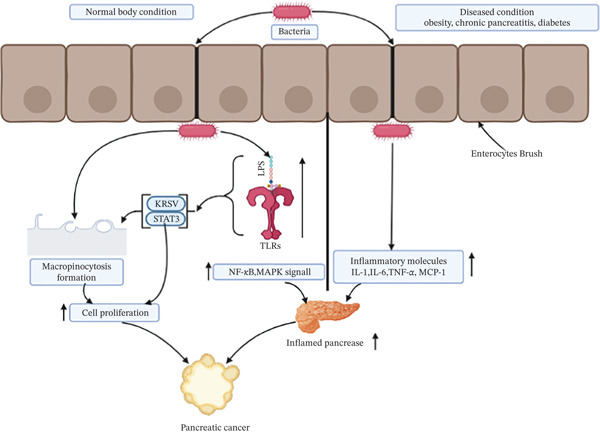
Potential role of bacteria in pancreatic cancer development. Pancreatic cancer starts with multiple changes, including bacterial metabolites like LPS, leading to the activation of NF‐*κ*B and MAPK pathways via TLRs. Besides, bacteria in patients with cardiovascular diseases activate some inflammatory molecules, such as IL‐1, IL‐6, TNF‐*α*, and MCP‐1, causing pancreas inflammation. Furthermore, bacteria can cause micropinocytosis and generate tumors‐promoting microenvironment, resulting in cancer progression.

Patients with obesity [95], chronic pancreatitis [96], and diabetes, due to the general survival of PC cells, have an increased risk of developing PC. In patients with these comorbidities, the innate immune system is activated through abnormal regulation of the NF‐*κβ* signaling pathway, leading to the induction of an immune response and inflammation. These inflammatory molecules secreted from adipose tissue include IL‐1, IL‐6, TNF‐*α*, and monocyte chemoattractant protein‐1 (MCP‐1) [97]. It has been shown that certain *Salmonella* species can induce actin remodeling‐mediated macro‐pinocytosis in human cells, which triggers the Wnt (Wingless/Integrated) signaling pathway and promotes cell proliferation and differentiation during carcinogenesis [98]. The role of *H. pylori* infection in PC is mediated by two plausible hypotheses that describe its role in pancreatic carcinogenesis [99]. For example, colonization of *H. pylori* in the antrum dramatically slows the central delta cells (*δ*‐cells), suppressing somatostatin (growth‐inhibitory hormone) production; consequently, S cells (cells that secrete secretin) are upregulated, increasing secretin and pancreatic bicarbonate production [100, 101]. These molecules positively affect DNA synthesis in pancreatic duct cells, which induces enhanced proliferation, leading to PC development. In addition, *H. pylori* can grow in the gastric corpus mucosa (which contains gastric glands) and can be associated with atrophic gastritis, leading to reduced HCl production, known as hypochlorhydria [102]. Consequently, pH is increased, which promotes bacterial growth. These bacteria increase N‐nitrosamine secretion, which can serve as a carcinogen and is associated with the pathogenesis of PC [103, 104]. A description of the graphic depiction of PC caused by bacteria is shown in Figure 3.

#### 3.2.4. GC

GC is one of the most common cancers and significantly contributes to global health [105]. It accounts for more than 1 million cases annually, about 5.7% of all cancers diagnosed worldwide [106]. Risk factors for this cancer include gastric ulcer [107], gastroesophageal reflux [108], obesity [109], cigarette smoking, alcohol consumption, high BMI, previous gastric surgery, exposure to chemical carcinogens, asbestos, heavy metals [110], and high salt consumption [111]. Furthermore, bacterial infections, especially *H. pylori* infection, which is the primary causative bacterial infection, are associated with the pathogenesis of GC [112, 113], although numerous microbes are available in the human stomach [114, 115]. However, a few bacteria, including *H. pylori* [116, 117], *Raoultella planticola* [118], *P. acnes*, *P. copri* [119], *Veillonella*, *Clostridium*, *Haemophilus*, *Staphylococcus*, *Neisseria*, and *Lactobacillus* [120], could contribute to the pathogenesis of GC.

However, the precise mechanism by which bacterial infections generate stomach cancer remains unclear. There are two mechanisms in which *H. pylori* infection promotes GC pathogenesis: *H. pylori* infection has two main effects on gastric epithelial cells: (i) it induces inflammation by indirect action and (ii) it directly affects the cells, inducing gene mutations and protein modification. These combined factors encourage the development of stomach cancer [121, 122]. *H. pylori* infection activates the host immune response [123] and helps to recognize pathogen‐associated molecular patterns (PAMPs) [124]. In turn, PAMPs trigger the production of inflammatory cytokines such as interferon (IFN)‐*γ*, IL‐1, IL‐2, IL‐6, IL‐8, IL‐12, and TNF‐*α* via activating TLRs, NF‐*κ*B, interferon regulatory factors (IRF), and activator protein 1 (AP‐1) [125, 126]. Also, the release of inflammatory mediators during the innate immune response activates T helper (Th)1/Th17 cell responses and stimulates the production of IFN‐*γ*, IL‐17, and TNF‐*α* [127]. These inflammatory responses are associated with the loss of acid‐secreting parietal cells, resulting in an elevation of stomach pH [128], and enhanced the production of ROS and RNS. These reactive species induce DNA single‐strand and double‐strand breaks, inhibit apoptosis and autophagy, and activate abnormal signals in gastric epithelial cells [129–131]. In addition, *H. pylori* infection correlated with cytotoxin‐associated gene (CagA), containing CagPAI and VacA, which in turn is directly involved with precancerous gastric lesion formation that can transform into the malignant phenotype [132]. CagA‐infected cells produce inflammatory products that lead to the risk of GC in multi‐steps, such as gastritis, intestinal metabolism, and dysplasia. Also, CagA induces the MAPK cascade leading to activation of Ras, RAF, E‐MEK, and ERK 1/2 in gastric epithelial cells, which trigger GC carcinogenesis by causing inflammation, cell proliferation, disrupting cell‐cell junctions, and inhibiting apoptosis [133–136]. VacA is another bacterial toxin that exhibits a poisonous effect on gastric epithelial cells and is associated with GC pathogenesis [137]. This toxin suppresses immune cell activation and proliferation. It also stimulates mast cell production of pro‐inflammatory cytokines (e.g., TNF‐*α* and IL‐6) to promote the development of *H. pylori*‐associated gastritis and peptic ulcers, which in turn promote GC pathogenesis [138, 139]. *H. pylori*’s CagA and VacA cooperated to degrade Janus kinase/signal transducers, which in turn activated JAK/STAT and NF‐ˈ*β*, causing inflammation and starting the carcinogenic pathways [140]. Additionally, these toxins may result in decreased tight junction function, increased cell invasion, pro‐inflammatory cytokine production (IL‐4, 6, 10, 11, TNF‐*α*), STAT‐3 activation, and NF‐*κ*β activation, all of which may promote the growth and survival of GC cells [141–143]. Moreover, in a Src‐EGFR‐dependent pathway, PGN from bacterial origin may stimulate PI3K‐PKB/Akt signaling via NOD1‐dependent activated NF‐*κβ* to initiate cell proliferation, migration/metastasis, and anti‐apoptosis pathways [35, 112]. Progression of GC has also been linked to a mutation in the D1 domain of Helicobacter’s flagellin F1aA, which helps avoid TLR‐5 recognition [144, 145]. CagPAI+, another bacterial factor, can increase gene mutation, especially in *p53*, by causing dysregulation of cell cycle control and death in gastric mucosal cells via the activation of the enzyme activation‐induced deaminase (AID) [146]. Gunathilake et al. reported that *P. acnes* and *P. copri* were more prevalent in patients with GC compared with controls, indicating that the existence of these species raises the incidence of GC [147]. By activating the natural killer group 2 member D (NKG2D) system and secreting the pro‐inflammatory cytokine IL‐15, *P. acnes* may cause corpus‐dominant lymphocytic gastritis [119]. On the other hand, *P. copri* produces the redox protein thioredoxin and enhances resistance to host‐derived ROS, which contributes to its pro‐inflammatory role in a number of illnesses [148]. Furthermore, by inducing the synthesis of NOCs, a number of bacteria, including *Nitrospirae*, *Lactobacillus*, *Haemophilus*, *Staphylococcus*, *Neisseria*, and *Ventobacillus*, contribute to the development of GC [149, 150]. These bacteria enter the epithelial cells of the gastric gland and cause inflammation, along with increasing the production of NO_2_ and NO_3_ by enhancing nitrate and nitrite reductase activities in chronic gastritis, resulting in the promotion of GC pathogenesis [151–153].

#### 3.2.5. LC

LC, which accounts for 11.4% of all cancer cases worldwide and will cause about 2,206,771 new cases and 1,796,144 deaths in 2020, is a danger to global health [1]. Recent research has revealed the existence of pathogenic bacterial species such as *Pseudomonas*, *Streptococcus*, *Staphylococcus*, *Veillonella*, *Moraxella* [154], *C. pneumonia*, *Streptococcus mitis*, *Staphylococcus epidermis, Bacillus* sp. *Mycoplasma sp.* [155] is correlated with the development of LC.

**Figure 4:**
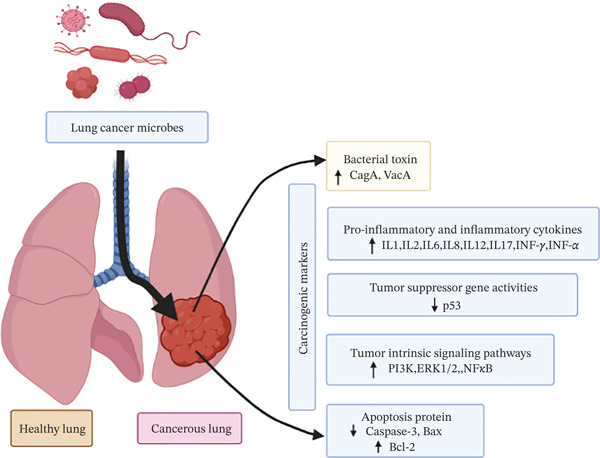
Hallmarks and interaction between bacterial infection and lung carcinoma. The sequential mechanism generates the tumor microenvironment in the lung by bacteria. Oncogenic bacteria secrete toxins, such as CagA and VacA; pro‐inflammatory cytokines, for example IL‐1, IL‐2, IL‐6, IL‐8, IL‐12, IL‐17, and tumor necrosis factor‐alpha (TNF‐*α*) and INF‐*γ*. Lung bacteria induce apoptosis by suppressing p53, caspase‐3, and Bax and activating the anti‐apoptotic protein Bcl‐2. Tumor intrinsic signaling pathways, including ERK, PI3K and NF‐*κ*B pathways, are triggered by bacteria and generate lung cancer.

The mucosal tissue of the lungs is home to a complex bacterial species, which, by activating T cells, has a close connection to the inflammation brought on by lung adenocarcinoma [156]. Certain beneficial bacteria induced the release of pro‐inflammatory chemicals, such as IL‐1b and IL‐23, which resulted in the expansion and activation of Vg6 + Vd1 + *γδ* T cells [157]. *C. pneumonia* stimulates lung carcinogenesis by the secretion of cytokines such as IL‐6, IL‐8, and TNF [158]. Moreover, this bacterial infection causes nitric oxide production and influences inflammatory reactions, promoting LC [159]. For example, ROS produced, followed by *M. tuberculosis* infection, could bind with DNA and induce genetic alterations associated with lung carcinoma [160]. Also, inflammatory cytokines including IL‐1, IL‐2, IL‐17, and IL‐12, tumor necrosis factor‐alpha (TNF‐*α*), and INF‐*γ* were released in *M. tuberculosis* infection, which could promote lung tissues carcinogenesis by activating the anti‐apoptosis mechanism and by suppressing tumor suppressor protein p53, apoptotic molecules caspase‐3, and Bax with increasing anti‐apoptotic protein Bcl‐2 expression [93, 160, 161]. By inhibiting the immune system, *Cryptococcus* spp. infection has also been connected to the progression of LC [162]. Infection with *H. pylori* may indirectly raise the risk of LC by secreting toxins such as CagA and VacA, and modulating Src/130cas signaling cascade [163]. These toxins release IL‐6 and IL‐8, which promote tumor properties, including cell proliferation, migration, and invasion through activating the NF*κ*B‐1 pathway. Furthermore, infection with *Haemophilus influenza* may increase the likelihood of tumor development in the lung mediating via IL‐17 [164]. In addition, bacterial species such as *Veillonella*, *Prevotella*, and *Streptococc*us are considerably associated with activating ERK and PI3K pathways in lung cells, initiating carcinogenesis [165]. Also, infection with *Acidovorax* in the lung causes mutations of tumor suppressor protein, including TP[53], which can cause LC pathogenesis [166, 167]. The graphic depiction of LC caused by bacteria is summarized in Figure 4.

#### 3.2.6. Gallbladder Cancer

**Figure 5:**
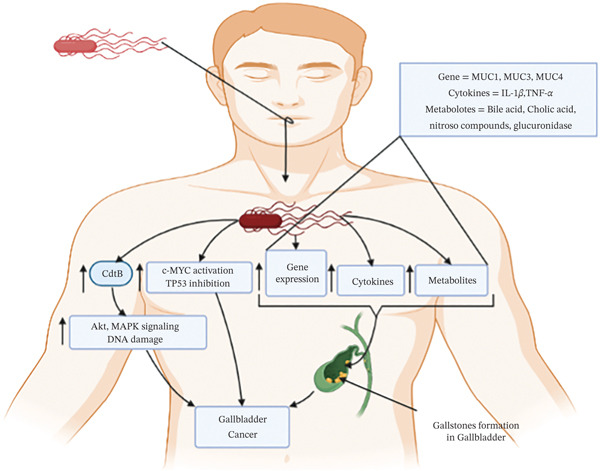
Mechanism of human gallbladder cancer formation by bacterial infections. TP^53^ and c‐MYC genes are the common genes activated by bacteria during gallbladder cancer pathogenesis. Bacteria in the gallbladder can produce toxins, like CdtB, CNF1, and mutagenic metabolites, including 5‐ alpha, 6‐alpha epoxide cholesterol, cholic acid, nitroso compounds, and a glucuronidase. CdtB toxin causes activation of MAPK and Akt‐protein kinase pathways, and bacteria causes activation of mucin genes, including MUC1, MUC3, and MUC4 are responsible for gallstone formation and finally gall bladder cancer.

Gallbladder cancers are a less common type of cancer, with an annual 4400 deaths worldwide [1]. Risk factors include chronic gall bladder infections, reproductive factors, obesity, malnutrition, and environmental exposure to chemical carcinogens, which are associated with gallbladder carcinogenesis [168, 169]. In addition, a chronic bacterial infection in the gallbladder might contribute to cancer genesis [170]. Mutations in TP53 and c‐MYC caused the malignant transformation of mouse gallbladder organoids and fibroblasts, followed by *Salmonella typhi* infections [171]. It was noted that *S. typhi* infection could produce toxic substances such as cytolethal distending toxins (CdtB) and cytotoxic necrotizing factor 1 (CNF1). CdtB induced DNA damage, whereas CNF1 modifies Rho proteins to influence the transcription termination process, resulting in gallbladder cancer [172]. Furthermore, CdtB could produce specific mutagenic metabolites that stimulate MAPK and Akt‐protein kinase pathways that promote and maintain cancerous transformation [173]. Additionally, bacterial infection can transform cholesterol into carcinogenic compounds such as 5‐ alpha, and 6‐alpha epoxide cholesterol and bile acids into mutagenic cholic acid, which can stimulate gallbladder cancer formation [174]. Apart from cholic acid, additional by‐products generated by *S. typhi*, such as nitroso compounds, glucuronidase, and secondary bile salts, are crucial in the occurrence of gallbladder cancer because they lead to the development of gallstones [175]. Through immunomodulation and modifications to mucin gel accumulation, the nucleation matrix that forms cholesterol gallstones in the gallbladder—a subset of microbiota modifies the expression of mucin genes, including MUC1, MUC3, and MUC4 [176, 177], and this gallstone increases the risk of gallbladder cancer‐related mortality [178]. Also, *H. pylori* infection increases inflammatory molecules such as IL‐1*β* and TNF‐*α* and activates the gallbladder’s tumor promoter genes, enhancing cell proliferation and invasion through autocrine mechanisms, resulting in the pathogenesis of gallbladder carcinogenesis [179]. A summary of the graphic depiction of gallbladder cancer brought on by bacteria is shown in Figure 5.

#### 3.2.7. OC

**Figure 6:**
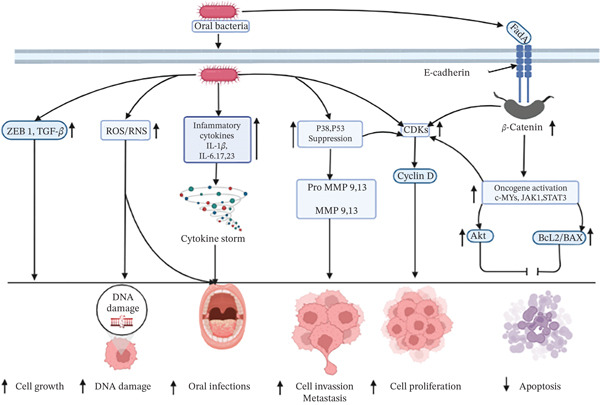
Possible diagram of the role of oral bacterial infection in oral cancer formation. Extracellular bacteria secrete FadA, activating the *β*‐catenin signaling and sequentially causing oncogenes like *MYC*, *JAK1*, and *STAT* activation. These oncogenes simultaneously inhibit apoptosis by activating anti‐apoptotic signaling, including Akt and Jak‐stat pathways, with increasing Bcl2/Bax ratio. Intracellular bacteria activate tumor suppressor proteins p38 and p53, which elevate MMP‐1 and MMP‐9 and regulate CDK to enhance cell proliferation and inhibit apoptosis. Moreover, bacteria cause cytokine storms via activating inflammatory cytokines, such as interleukin 1*β* (IL‐1*β*), IL‐6, IL‐17, IL‐23, and TNF‐*α* and damage DNA by activating oxidative stress, resulting in oral carcinoma.

OC’s morbidity and death represent a global health burden [180]. Age, malnourishment, genetics, family history, radiation and X‐ray exposure, alcohol use, tobacco use, and smoking are risk factors for OC [181] along with oral dysbiosis associated with the pathogenesis of oral carcinoma [182]. The human oral microbiome has a profound impact on health, and microbe–host imbalances may cause systemic and oral diseases as well as chronic inflammation, which can accelerate the development of cancer [183]. Inflammatory mediated oral carcinoma can be caused by oral bacteria such as *Porphyromonas*, *Prevotella*, and *Fusobacterium* spp., which are classified as periodontal microbes. These bacteria can induce the secretion of different inflammatory cytokines, including interleukin 1*β* (IL‐1*β*), IL‐6, IL‐17, and IL‐23, TNF‐*α*, and metastatic proteins MMP‐8 and MMP‐9. Also, bacterial species in the oral cavity have generated ROS and RNS species [184]. These reactive molecules are associated with the free radical generation, interact with chronic inflammation, and are related to cancer development [185, 186] through damaging genomic DNA [187]. *P. gingivalis* infection results in the release of gingipains, which in turn trigger the activation of protease‐activated receptor (PAR). This process produces promatrix metalloprotease 9 (pro‐MMP 9) and transforms pro‐MMP‐9 into MMP‐9 [188]. Moreover, inside the host cell, *P. gingivalis* inhibits apoptosis after infection by activating anti‐apoptotic signaling, including Akt and Jak‐stat pathways [189, 190] with increasing Bcl2/Bax ratio [191]. Additionally, *P. gingivalis* can accelerate the cell cycle’s progression by modulating the S phase by altering cyclin and its associated enzyme cyclin‐dependent kinase (CDK) activity via suppressing P53 activity [192]. Oncogenic interactions between bacteria and oral epithelial cells may develop through *F. nucleatum* infection, followed by *β*‐catenin signaling pathway activation, resulting in increased cell survival and cell proliferation. For example*, F. nucleatum* infection is associated with enhanced activity of the cell cycle regulatory enzyme CDK and decreased tumor suppressor protein p38 with the production of MMP‐9 and MMP‐13 [66]. Also, *Fusobacteria* modulate and promote oral carcinoma through upregulating several metastatic factors such as MMP‐1, MMP‐9, and IL‐8, tumor promoter MYC, JAK1, and STAT3, along with epithelial–mesenchymal transition factors ZEB1 and TGF‐*β* [66]. Moreover, several common oral bacterial species can produce lactic acid [193], H_2_S, acetaldehyde [194], etc., which may develop an acidic and hypoxic tumor microenvironment, thereby increasing metastatic properties and progression of oral carcinomas [194–197]. A summary of the graphic depiction of OC brought on by bacteria is shown in Figure 6.

#### 3.2.8. Prostate Cancer (PCa)

Because of its high incidence rate and status as the primary cause of cancer‐related fatalities, PCa has caused substantial concerns for public health worldwide [198, 199]. PCa pathogenesis may be initiated by several variables, including age, family history, ethnicity, lifestyle, and occupational and environmental carcinogen exposure [200, 201]. However, human microbiota, especially urinary and gut microbes, might be a possible cause of PCa risk.

**Figure 7:**
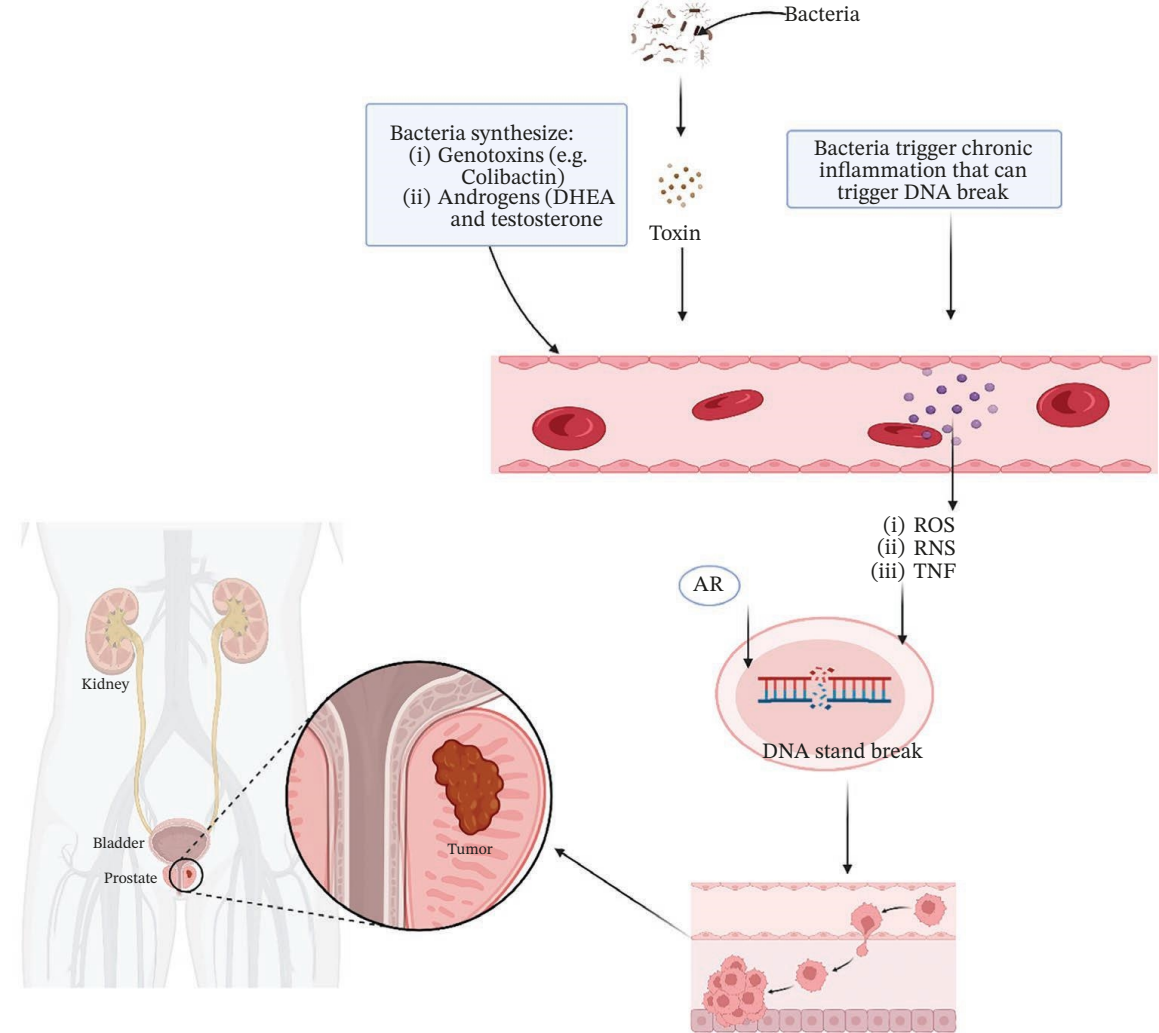
Bacteria contribute to prostate cancer development by synthesizing genotoxins (e.g., colibactin) and androgens (e.g., DHEA, testosterone), inducing chronic inflammation and oxidative stress (ROS, RNS, TNF), leading to DNA strand breaks. This mechanism promotes tumor formation in organs like the bladder and prostate, highlighting bacterial‐induced carcinogenesis pathways.

The development of PCa has been related to prostate inflammation via the modulation of many potential pathways [202]. For instance, bacterial inflammation triggers immune cells to generate ROS and RNS, which may directly damage DNA and result in genetic instability in epithelial cells [203–205]. Consequently, proliferative inflammatory atrophy (PIA) zones were created as a result of an increase in the proliferation of atrophic luminal epithelial cells [204]. In addition, a variety of inflammatory cytokines is released by the extracellular matrix during inflammation, such as TNF, IL‐7, IL‐2, RANTES, and MIP‐1. These cytokines help to secrete growth factors, such as fibroblast growth factors and transform growth factor‐*β*, which stimulate the growth of surrounding stromal cells and cause prostate hyperplasia and metastasis [206]. Additionally, they disrupt DNA and promote angiogenesis, cytoskeleton remodeling, and ECM degradation, which provide the right conditions for the establishment and development of cancer [206]. Furthermore, higher quantities of free estrogens are released into circulation due to *β*‐glucuronidase deconjugation activity caused by *β*‐glucuronidase production by the gut microbiota. These hormones induce DNA to become apurinic, which leads to mutations and oncogenesis [207]. Moreover, *E. coli* can attach itself to epithelial cells by adhering to carcinoembryonic antigen‐related cell adhesion molecules through Afa/Dr adhesion molecules. This results in hypermethylation of the CDK inhibitor 2A gene in urothelium cells, which in turn promotes the pathophysiology of PCa [208–210]. A summary of the graphic depiction of OC brought on by bacteria is shown in Figure 7.

#### 3.2.9. Ovarian Cancer (OVC)

Reproductive cancer, or OVC, is a leading cause of cancer‐related mortality for women globally, accounting for 13,770 fatalities and 21,410 new cases per year [199]. Although there is a connection between genetic and other factors with OVC, the actual cause is yet to be established. Recent research on microbe‐mediated carcinogenesis reported that bacterial infection has a significant role in developing OVC.

**Figure 8:**
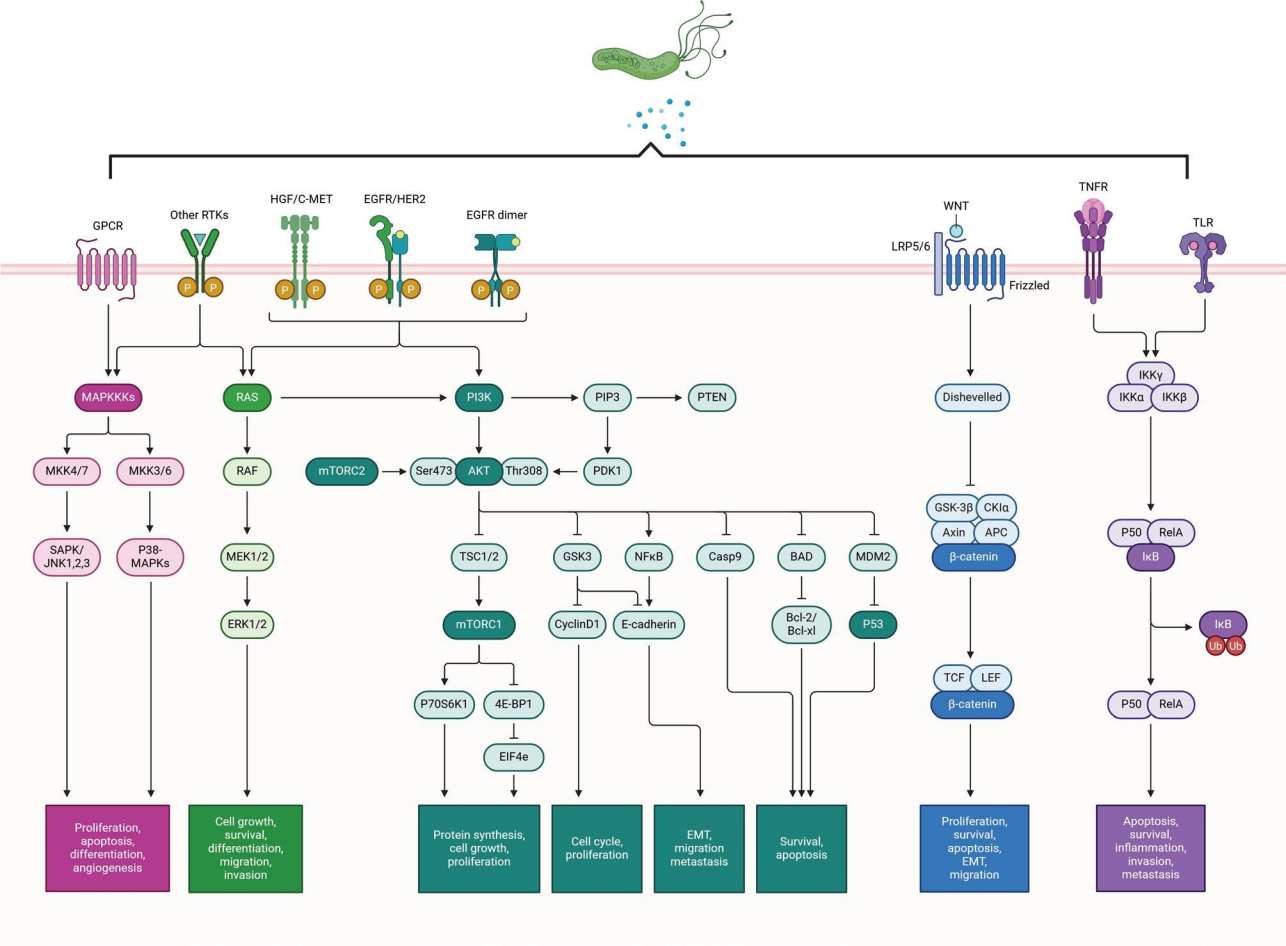
The schematic illustrates the signaling pathways activated by bacteria, contributing to gastric cancer progression. Key pathways include MAPK, PI3K/AKT/mTOR, WNT/*β*‐catenin, and NF‐*κ*B, each mediating cell proliferation, survival, EMT, invasion, and metastasis. Receptor tyrosine kinases (RTKs) and Toll‐like receptors (TLRs) initiate these cascades, influencing tumor microenvironment and immune modulation.

The exact mechanism of bacterial infection‐induced OVC is not precise. Nevertheless, bacteria or their metabolites may operate locally or penetrate the host’s systemic circulation to produce effects that are similar to hormones, autocrine, and paracrine. For instance, LPS could stimulate EMT and cancer cell migration growth‐promoting phenomena via activating PI3K signaling and increasing N‐cadherin, Slug, Vimentin, Snail, *α*‐SMA, TCF, MMP2, and MMP9 expression [211]. Lysophosphatidic acid is another metabolite that alters OVC cells’ activity by affecting several cancer‐related pathways. Through the upregulation of Akt, MAPK, and calcium signaling, they enhance cell motility, invasion, and proliferation [212, 213]. Also, pathogenic bacteria can promote the carcinogenesis of OVC via modulating host immune components. For example, infection of *Chlamydia trachomatis*, a gram‐negative, obligate bacterium, can activate innate and adaptive immune systems via regulating the secretion of cytokines such as IFN‐*γ*, IL‐6, IL‐8, IL‐10, and IL‐12 [214]. In addition, the roles of *Mycoplasma genitalium* in OVC were uncertain. However, researchers have shown that infection with this bacterium causes ovarian tumors [173, 215]. An infection with *M. genitalium* is linked to an increase in immune components such as TLR agonist‐stimulated chronic inflammation, granulocyte colony‐stimulating factor, granulocyte/macrophage colony‐stimulating factor (GM‐CSF), and monocyte chemotactic protein (MCP)‐1, as well as inflammatory cytokines (IL‐6, IL‐8) [173]. Moreover, infection with this bacterial strain produces a toxin that causes swelling of the tubal epithelial cilia, leading to an inflammation‐mediated formation of OVC [215]. A summary of the graphic depiction of OVC brought on by bacteria is shown in Figure 8.

### 3.3. Possible Mechanism of Phytochemicals Against Bacterial‐Induced Cancer Prevention

Over the years, conventional cancer treatments, including radiation, chemotherapy, and surgery, have seen remarkable improvements in survival rates [216]. However, the practical uses of traditional chemotherapeutics are limited because of their limitations, which include the development of resistance to them, recurrence of cancer after treatment, induction of secondary malignancies in patients with metastatic disease, and other adverse effects [217]. Thus, novel strategies such as chemo‐agents of natural origin could provide better efficacy with less or no toxicity to the non‐neoplastic cells, therefore, could better manage patients with cancer.

Nature‐derived drugs, including herbs for therapeutic purposes against bacterial infection‐mediated cancer, may be an innovative approach to get around the drawbacks of traditional chemotherapy, thus offering successful cancer treatment. The natural products may be utilized as nutritional supplementation (Table 1) or supplemented with conventional treatments (Table 2). Herbal‐based management of cancer includes either consumption as a dietary food before the infection prevents bacterial infection or consumption of specifically tested phytochemicals that can target pathways, or their components involved in cancer pathogenesis.

**Table 1:** Summary of phytochemicals against bacterial‐induced cancer in various models.

SL. no	Phytochemical	Structure	Sources	Cancer types	Observed anticancer mechanism	Ref
1.	Catechin		Green tea	Breast, prostate	↑Cell cycle at the G2 phase ↓Oxidative stress ↓STAT3‐NF*κ*B and PI3K/AKT/mTOR pathways	[218–220]
2.	Benzyl isocyanate		Alliaria pilu‐ oil, papaya seeds, petiolata,	Breast, prostate, pancreatic, colon	↑G2/M cell cycle arrest ↓MMP‐2/9, PKC, and MAPK signaling ↓PI3K/AKT/FOXO pathway, STAT3 ↓HIF‐1*α*/VEGF/Rho‐GTPases inhibition	[221–223]
3.	Capsaicin		Chili pepper	Pancreatic	↓ AP1, NF‐*κ*B, and STAT3 signaling, cell cycle arrest, ↓ *β*‐catenin signaling	[224]
4.	Lycopene		Tomatoes, papaya, grapefruit, guava, carrot	Prostate, breast	↓NF‐*Κ*b, Wnt‐TCF signaling	[225, 226]
5.	Epigallocatechin gallate		Green tea	Breast, pancreatic, lung	↓P13K/AKT and Telomerase ↑ Bax/Bcl‐2, CASP3, CASP9 and PTEN ↑ FKHRL1/FOXO3a ↓MMP‐2/9 protein and AMPK signaling ↑ p53, p21, p16 and Rb	[227–230]
6.	Genistein		Soy milk and soy‐based beverages	Breast, prostate, gallbladder	↑ p21 p21WAF1, p53 ↓ PLK‐1 expression, cyclin B1 ↓ Estrogen receptor (ER) *α*, disabled homolog 2 (DOC 2) ↑Arrest at G2/M	[231–233]
7.	Cucurbitacin B		Cucurbitaceae family	Colorectal, lung, breast, pancreatic	Inhibitors of JAK‐STAT3 MAPK signaling pathways	[234, 235]
8.	Kaempferol		Spinach and kale, herbs,	Prostate, breast, lung	↑GM‐GSC, PLC, PKC, MEK1/2 activation ↓MAP kinase ↑PARP cleavage, Bax, and p21 ↓Bcl‐2, cathepsin D, cyclin E, and cyclin D1 ↑ miR‐340, PTEN ↓p‐PI3K and p‐AKT	[236–238]
9.	Lutein		Broccoli, spinach, peas, lettuce, and egg yolks	Prostate, breast, colorectal	↓ ERK, AKT/mTOR AKT/ERK/mTOR, P70S6K signaling, MMP‐2,‐9 ↑p53 and Bax ↓Antiapoptotic gene, Bcl‐2 ↓ K‐ras, *β*‐catenin, ERK1/2 and PKB	[239–241]
10.	Naringenin		Fruits like citrus species and tomatoes	Prostate, breast, pancreatic	↑ Bax/Bcl‐2 ratio ↓AKT/ERK1/2, P70S6K, S6, P53, P38, and JNK ↓E‐cadherin ↓P13K, MAPK, ERK1/2, AKT ↑ ASK1, JNK, p38, and p53	[242–245]
11.	Sesamin		Vascular plants, sesame seed, leaves	Prostate, lung cancer	↓Surviving, Bcl2,COX 2,Cyclin D1 ↓MMP‐9, ICAM‐1,VEGF, TGF‐*α*, IL‐6 ↑ G0/G1 phase arrest ↑Caspase‐3 and cleaved caspase‐9, PINK1	[246–248]
12.	Hesperetin		Citrus fruits, including oranges, grapefruit, and tangerines	Colon, lung pancreas, oral, breast	↑JNK‐1, JNK‐2 ↑Caspase9, caspase3 IL‐1*β*, CDK ↑p21, p27 ↓COX‐2, MAPK, ERK1/2, NF‐*κ*Bp65 expression ↓PGE2 expression	[249]
13.	Wogonin		Scutellaria radix	Breast, gallbladder	↑HIF‐1*α* degradation, ↓HIF‐1*α* protein aggregation and translation ↓Hsp90 client proteins EGFR, Cdk4, and surviving ↓MMP‐2, MMP‐9 ↑phosphorylation of ERK1/2	[250, 251]
14.	Fisetin		*Fragaria ananassa, Malus domestica* (Fruit)	Breast, colorectal, pancreatic	↓ P13K, MAPK, STAT ↓OP2A, KIF20A, CCNB2 and CCNB1 ↑CDKN1A, SEMA3E, GADD45B and GADD45A	[252, 253]
15.	Formononetin		Beans, soy, and other vegetables	Prostate, lung, colorectal	↑Arrest at G0/G1 phase ↓ cyclin D1,E CDK4, and Akt/↓ ERK1/2, MAPK activity ↑ BCL2‐associated X (Bax) ↑ p53 expression, miR‐149 EphB3, p‐AKT, p‐P13K,p‐STAT3, cyclin D1, MMP2/9	[254–257]
16.	Quercetin		Nuts, apples, onions, olive oil, broccoli, red grapes	Prostate, breast, oral carcinoma	↑Mitochondrial integrity, ROS, Raf/MEK signaling ↓ Pro‐survival Akt pathway ↓Wnt, *β*‐catenin ↓‐kB, COX2, and MMP9 miR‐22	[258–260]
17.	Glycitein		Soy food products	Prostate, gastric	↓ERK1/2, VEGFR ↑Arrest G2/M phase ↑Halt MAPK/STAT3/NF‐*κ*B ↑G0/G1 phase arrest	[261–263]
18.	Rhaponticin		Rheum rhaponticum, rhubarb species	Breast	↓ VEGF level	[264]
19.	Esculin		Trees: horse chestnut, California	Pancreatic, lung	↑ Nrf2, p27 and p21 ↓NF‐*κ*B, Sp1	[265, 266]
20.	Isorhapontigenin (ISO)			Pancreatic, gastric, colon, lung adenocarcinoma	↑ Caspase‐3 activation	[267]
21.	Biochanin A		Red clover, soy, peanuts,	Pancreatic, oral	↓ Akt and MAPK, and Akt	[268, 269]
22.	Isoflavone		Soy, lentils, beans, and chickpeas	Gastric, breast, prostate, lung	Inhibition of c‐erB‐2, MMP‐2, and MMP‐9 signaling pathways, Affecting IGF‐1R/p‐Akt signaling transduction	[270]
23.	Piperlongumine		Roots of long pepper	Pancreatic, colon cancer, oral, breast	Autophagy‐mediated apoptosis by inhibition of PIK3/Akt/mTOR	[271]
24.	Resveratrol		Grapes, peanuts, and soy	Breast, colon, prostate, breast	↑p21CIP1/WAF1 ↓Akt phosphorylation ↑Activate procaspase‐9 ↓NF‐*Κ*b,Cox2, MMP9	[272]
25.	Luteolin		Parsley, onion leaves, carrots, peppers, cabbages	Colon, lung	↓PI‐3‐Kinase/Akt ↑AI‐P, p21, caspase 3, Bax/Bcl2 ratio	[273, 274]
26.	Apigenin		Parsley, chamomile, celery, oregano	Colorectal, lung, pancreatic	↓Wnt/*β*‐catenin signaling ↓FAK, Src, and Akt phosphorylation, ↓Cyclin A, cyclin B, and CDK1 ↓ PI3K/Akt ↑Ikaros expression	[275–278]
27.	Isoliquiritigenin		Licorice, soy beans, and shallots	Gastric, lung, and colon	↓p62,p‐AKT, p‐TO, PI3K/AKT/mTOR ↑Bax,caspase‐3 ↓Bcl‐2, mTOR,PI3K/AKT p ↓ p‐AKT, p‐mTOR, CyclinD1	[279–281]
28.	Coumestrol		Soybeans, clover, sunflower seeds, spinach, and legumes.	Breast, colon	↑ ROS production ↑ p53 and p21Cip1/WAF1 activity	[282]
29.	Psoralen		Limes and lemons, celery, parsley, figs, and cloves	Breast	↑ G0/G1 & G2/M phase arrest ↓ Fra‐1 & *β*‐catenin expression ↑ Axin2 & phospho‐(Y142) *β*‐catenin expression	[283]
30.	Delphinidin		Berries, purple sweet, potatoes, red cabbages	Lung	↓PI3K, Bcl2, Bcl‐xL and Mcl‐1, cyclin D1. ↓phosphorylation of AKT and MAPKs ↑ PARP protein, caspase‐3 and ‐9, Bax, and Bak	[284]
31.	Curcumin		Curcuma longa	Lung, gastric	↓Wnt/*β*‐catenin, VEGF, NF‐*κ*B, NOTCH 1, ERK1/2 ↓PI3K, Wnt3, a/*β*‐catenin, BCL‐2	[285, 286]
32.	Ursolic acid		Apples, bilberries, elderflower, rosemary, lavender, oregano,	Oral, gastric	↓Akt/mTOR/NF‐*κ*B signaling, ERK, MMP‐2 ↑p62, p38, miR‐133a ↓G1 phase arrest, Akt1	[287, 288]
33.	Sauchinone			Pancreatic, gastric	↓Hypoxia condition ↓Wnt/*β*‐catenin, TGF‐*β*1, I3K/Akt and Smad2/3 signaling	[289, 290]
34.	Oleuropein		Olive tree, fruits, leaves.	Pancreatic, lung	↑aspase 3/7 activation, Bax protein. ↓c‐Jun and c‐Fos ↓mitochondrial Glo2, Akt signaling pathway	[291, 292]
35.	Chrysin		Honey, propolis, flowers, and Passiflora incarnata	Oral, lung	↑Caspase‐3/7 ↓AKT and PI3K ↑Caspase 3, caspase 9, Bax/Bcl‐2 ratio	[293, 294]
36.	Calycosin			Pancreatic, colorectal	↑p21Waf1/Cip1 ↓Epithelial‐mesenchymal transition, matrix metalloproteinase ↓ER*α*, IGF‐1R,p‐Akt,miR‐95	[295, 296]
37.	*α*‐mangostin		Bark and dried sap of Garcinia mangostana L	Gallbladder, lung	↓AMPK/SREBP1	[297, 298]
38.	Emodin		Chinese rhubarb	Colon	↑Caspases ↓MAPK/JNK, PI3K/AKT, NF‐*κβ* and STAT, Bcl‐2	[299]
39.	Ginsenosides		Ginseng	Breast, gastric cancer, and lung	↓Akt/mTOR/p70S6K, miR‐18a, Smad2, VEGF, G0/G1 phase arrest ↑Bax/Bcl‐2 ratio ↓Raf/MEK/ERK, AKT/mTOR, and AKT/GSK‐3*β*/*β*‐catenin signalling	[300–303]
40.	Jasmonates			Colorectal, lung	↓Survivin, ↑Bax, p21	[304, 305]
41.	Pterostilbene		Grape leaves, Pterocarpus marsupium heartwood	Pancreatic, oral, colorectal	↓MDR1 protein, Akt signal, Bcl‐2 ↑SOD2 expression	[306–308]
42.	Shikonin		The root of the plant Lithospermum erythrorhizon	Pancreatic, gallbladder, lung	↑RIP1 and RIP3 regulation, G0/G1 phase arrest, JNK signaling pathway, FOXO3a, Bim, EGR1	[309–311]
43.	Silibinin		Silybum marianum	Lung, colon	↓ERK1/2, Akt, Cyclins (D1, D3, A and B1) ↑CDKN1A (Cip1/p21), CDKN1B (Kip1/p27) ↓COX‐1, COX‐2, NOS, NOS3, VEGF	[312–315]
44.	Withaferin A		Ayurvedic medicine	Colon, lung, pancreatic	↓ IL‐6, STAT3, Bcl‐2, Akt. Hsp90 activity, Akt, Cdk4 and glucocorticoid receptor ↑Caspase‐3 and cleavage of PARP	[316–318]
45.	Equol		Dairy and egg products	Gastric	↓Ki67, G0/G1 cell cycle arrest, CDK2/4, Cyclin D1/Cyclin E1 ↑P21, cleaved PARP, and caspase‐3, P‐Akt	[319]
46.	Matairesinol		Beverages, vegetables, nuts, bread, and fruits	Prostate	↓HDAC8 activity ↑TRAIL, Cyt‐C release, caspase‐8, ‐3 ↓ AKT signaling	[320, 321]
47.	Baicalein		Scutellaria baicalensis, Indissan trumpet flower	Gastric, colorectal, lung	↓TGF‐*β*/smad4, PTEN, HIF‐*α* ZEB1/2,N‐cadherin,MMP2/9,Akt,NF‐KB,MMP2/9, TNF‐*α*, IL‐*β*, iNOS, COX‐2, ↑P53, RUNX3 and FOXO3a	[322]
48.	Phloretin		Apple tree leaves and Manchurian apricot.	Gastric, lung	↑G2/M phase arrest, Bcl‐2, MMP‐2 and ‐9 ↓p‐JNK and p‐p38 MAP, cleaved‐caspase‐3 and ‐9	[323, 324]
49.	Wedelolactone		Bhringaraj plant	Breast	↑ Chymotrypsin‐like activity ↑ ROS production ↑ p53 and p21Cip1/WAF1 activity ↓PKC*ε*	[325, 326]

**Table 2:** Summary of the synergistic mechanism of phytochemical with combination with anticancer therapeutics agents.

Phytochemicals	Treatment combination	Cancer type	Effect	Ref
1. Apigenin	Cisplatin	Breast Ovarian	↓Cell proliferation ↓Telomerase activity ↑Apoptosis ↓Metastasis	[327–329]
	Navitoclax	Colon	↓Tumor growth	[330]
2. Psoralen	Docetaxel	Lung	↑**U**ptake of chemotherapy ↑Drug sensitivity	[331]
3. Silibinin	Cisplatin	Breast	↑Apoptosis	[332]
	Erlotinib	Lung	↓Metastasis	[332]
	Mitoxantrone	Prostate	↑Apoptosis	[332]
4. Daidzein	Topotecan	Breast	↑Cell viability	[333]
	Epirubicin	Colon	↑ROS levels ↑Cell cycle arrest ↑Apoptosis	[334]
5. EGCG	Docetaxel Paclitaxel	Prostate Breast	↑Drug sensitivity	[335, 336]
	Gemcitabine	Pancreatic	↑Drug sensitivity	[337]
6. Equol	Tumor‐associated macrophage	Breast	↑Drug sensitivity ↑Apoptosis	[338]
7. Formononetin	Sunitibib	Breast	↑Cell cytotoxicity	[339]
8. Genistein	Tamoxifen	Breast	↓Cell viability	[340]
	Cisplatin	Gastric and Pancreatic	↑Drug efficacy	[341, 342]
	Docetaxel	Pancreatic	↓NF‐*κ*B signaling ↑Trigger apoptosis	[341]
9. Luteolin	Doxorubicin	Breast	↑Drug efficacy ↑Antioxidant capacity	[343]
10. Quercetin	Doxorubicin	Breast	↓HIF‐1*α* and P‐gp expression	[344]
11. Resveratrol	Cisplatin	Colon	↑Oxidative stress	[345]
	Etoposide	Colon	↓Cell viability	[345]
	Oxaliplatin	Colon	↑Anti‐tumor activity	[346]
	Gemcitabine	Pancreatic	↑Drug sensitivity	[347, 348]
	Sorafenib	Breast	↑ROS production, ↑Cell cycle arrest, ↑Caspase 3 and PARP cleavage	[349]

Natural substances have a more prominent effect on cancer cells [350] than non‐neoplastic ones, as they can induce apoptosis, either activation of intrinsic or extrinsic pathways, thereby interfering with carcinogenesis and inhibiting tumor growth and development. For example, a group of phytochemicals including Epigallocatechin gallate (EGGG) [227], Kaempferol [236], Lutein [239], Esculetin [265], Naringenin [242], Sesamin [246], Formononetin [254], Quercetin [258], Biochanin A [351] etc. impede carcinogenesis via induction of apoptosis in several cancers. The underlying mechanism is to increase caspase‐related enzymes, for example, caspase 3, 7, 9, 8, PARP cleavage, apoptotic protein Bax, and decrease anti‐apoptotic protein Bcl‐2, along with activating tumor suppressors such as p21, p27, p38, p53, and p62, followed by phytochemical treatments. Also, phytochemicals significantly inhibited cancer cell proliferation in various cancers by suppressing signaling molecules and cell cycle regulatory proteins. For example, Cucurbitacin B [234], Psoralen [283], Jasmonates [304], Formononetin [255], Glycitein [261], etc. act as potent inhibitors of the cell cycle in various cancers. Through either blocking cyclin expression or activity, they may stop the cell cycle from continuing (B1, D1, E) and their associating enzyme CDKs (4, 6, 7) or increasing cell cycle regulatory proteins p21, p27, p38, p53, and retinoblastoma protein (Rb).

**Figure 9:**
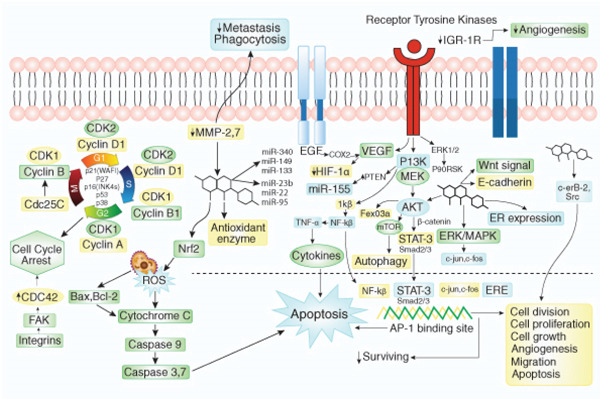
Role of phytochemicals in targeting bacteria‐induced carcinogenic pathways. Phytochemicals induce apoptosis through intrinsic and extrinsic pathways and mediate cell cycle arrest by modulating cell cycle regulatory protein. The vital pathways that phytochemicals target are PI3K/AKT and MAPK (ERK1/2). Several miRNA expressions followed by suppression of cell migration, invasion, and angiogenesis are also regulated by natural products to mitigate bacteria induced carcinogenesis.

Additionally, natural products exhibited antiangiogenic effects both *in vitro* and *in vivo,* thus, can act as angiogenesis inhibitors, which could block tumor cell growth, multiplication, and metastasis. For instance, phytochemicals, such as silibinin [312], Luteolin [240], Wogonin [250], Rhaponticin [264], and Lycopene [225], exhibited antiangiogenic properties via suppressing the expression of vascular endothelial growth factor, hypoxia‐inducible factor 1a (HIF‐*α*), and matrix metalloproteases (MMP 2, MMP 9). Furthermore, phytochemicals could modulate cell proliferation and survival signaling and act as potential chemotherapeutic agents by targeting cell‐signaling pathways or their components. For example, Benzyl isothiocyanate [221], Resveratrol [272], Glycitein [261], Apigenin [275], Delphinidin [284], Calycosin [295], Ginsenoside [300], etc. can target many signaling pathways such as NF‐*κ*B, PI3K/Akt signaling mechanism by suppressing IGF‐IR, Akt phosphorylation status, MAPK/ERK intermediate Ras, Raf, MEK, suppressing p‐65, PI3K and p‐mTOR and regulating Wnt/*β*‐catenin, Notch‐1, hedgehog signaling. Altogether, phytochemicals may have the potential to effectively eliminate cancer cells via targeting several cellular pathways or their components, thus providing better options for patients with bacterial infection‐induced carcinogenesis. Figure 9 illustrates the summarized effects of phytochemical‐mediated anticancer properties.

### 3.4. Nanotechnology‐Based Strategy on Bacterial‐Induced Cancer Treatment

Nanotechnology‐based interventions have emerged as promising strategies to target both bacteria and the cancer cells they influence, enabling precise, localized treatment with minimized side effects [352].

#### 3.4.1. Antimicrobial Nanoparticles

Metal‐based nanoparticles, such as those of silver, gold, and zinc oxide, exhibit potent antibacterial properties due to their ability to disrupt bacterial membranes, generate ROS, and interfere with bacterial DNA [353]. These nanoparticles are particularly useful in targeting *H. pylori*, which is a significant etiological factor in GC [354]. Bacterial biofilms pose a significant barrier to treating infections associated with cancer. Nanoparticles engineered with biofilm‐disrupting enzymes, such as DNase‐functionalized silver nanoparticles, can penetrate and dismantle biofilms, exposing bacteria to immune responses and antibiotics [355]. For instance, studies have demonstrated that silver nanoparticles can inhibit *H. pylori* growth and biofilm formation, decreasing the inflammatory response in gastric tissue [356–358]. Additionally, cell membrane‐coated nanoparticles (CNPs) are designed to target carcinogenic bacteria effectively, neutralizing their virulence factors and enhancing bacterial eradication [359]. CNPs can modulate antibacterial immunity, providing a multifaceted approach to combatting cancer associated with bacterial infections [360]. Such antimicrobial activity is crucial in reducing bacterial loads and consequently the risk of cancer progression.

#### 3.4.2. Targeted Drug Delivery Systems

Nanoparticles can also serve as carriers for antibiotics and chemotherapeutics, enabling targeted drug delivery directly to infected tissues [361]. Their unique physicochemical properties allow for enhanced penetration of biological barriers, leading to increased therapeutic efficacy while minimizing side effects. It is reported that nanoparticles can cross the blood–brain barrier, making them suitable for treating brain tumors and offering potential for addressing cancers associated with bacterial infections [362]. Nanoparticles can easily infiltrate tissues due to their nanoscale size, allowing for direct delivery to infected areas [363]. They can be engineered for controlled release, responding to specific biological signals, which ensures that drugs are released precisely when and where needed [364]. For example, polymeric and liposomal nanoparticles encapsulating antibiotics have shown enhanced therapeutic effects by localizing drugs to bacterial infection sites while minimizing off‐target toxicity [365]. In particular, liposomal delivery systems have demonstrated improved efficacy in treating *H. pylori*‐induced GC, where antibiotic encapsulation increases the drug’s half‐life and enhances its penetration into the gastric mucosa [366]. Recently, pre‐clinical investigation reported that potentials of nano‐based strategies to combat bacteria‐associated carcinogenesis. Membrane‐coated nanoparticles developed by Angsantikul et al. targeted *H. pylori* in mice, improving gastric retention and enhancing bacterial eradication compared with free antibiotics [367]. Similarly, Pinho et al. reported that cholesterol functionalized “Trojan horse” successfully entered *H. pylori* cells and markedly reduced infection levels in vivo [368]. Beyond gastric pathogens, Chen et al. showed that a *F. nucleatum* membrane–coated nano‐vaccine reduced bacterial colonization and suppressed colorectal tumor formation in murine models [369].

### 3.5. Immune Modulation

Nanoparticles can modulate immune responses to enhance bacterial clearance and inhibit cancer progression. For example, nanoparticles functionalized with bacterial antigens or DNA have been shown to stimulate a robust immune response, promoting recognition and elimination of bacteria‐related cancer cells [370]. Zhang et al. showed that combining nanoparticles with immune checkpoint inhibitors has shown to amplify immune responses, leading to improved tumor control and reduced metastasis [371, 372]. Additionally, these nanoparticles can serve as adjuvants, amplifying immune responses to bacterial vaccines specifically targeting cancer‐associated pathogens [373]. Nanoparticles can deliver bacterial antigens directly to dendritic cells, enhancing antigen presentation and T‐cell activation, as demonstrated with a nano‐vaccine targeting *F. nucleatum* in CRC [374]. Moreover, Xu et al. reported that biohybrid bacteria engineered with nanobodies can selectively target tumors, leading to effective tumor suppression and macrophage activation, which is crucial for anti‐tumor immunity [375].

### 3.6. Photothermal (PTT) and Photodynamic Therapy (PDT)

PTT and PDT use light‐activated nanoparticles, such as gold or graphene oxide, to produce localized heating or ROS upon laser irradiation [376]. This effect not only damages cancer cells but also targets bacterial pathogens residing within the tumor microenvironment [377]. Previously, it was shown that gold nanoparticles in combination with PTT have successfully reduced *H. pylori* load and tumor size in GC models, showcasing their dual antibacterial and antitumor effects [378, 379].

## 4. Conclusion and Future Prospective

Despite providing a comprehensive synthesis of current evidence, this study has several limitations that should be acknowledged. This review just relies on previously published data and does not include original experimental or clinical validation, which may limit causal interpretation. The heterogeneity of study designs, bacterial strains, host models, and cancer types across the literature makes direct comparison difficult and may introduce bias. Nevertheless, this review is important because it consolidates fragmented evidence linking bacterial infections to carcinogenesis across multiple organ systems and highlights shared molecular pathways such as chronic inflammation, DNA damage, and dysregulated signaling. By integrating mechanistic insights with emerging therapeutic strategies, including phytochemicals and nanomedicine, this work provides a conceptual framework for future translational research. Importantly, it underscores the microbiome–cancer axis as a modifiable risk. Emerging evidence indicates that phytochemical and nanotechnology‐based interventions may effectively counteract bacteria‐induced carcinogenic pathways, offering promising adjunctive or alternative therapeutic options. Future studies should focus on identifying specific bacterial strains involved in different cancers, clarifying host–microbe interactions, and evaluating targeted therapies in clinical settings. Integrating conventional treatment with phytochemical‐rich dietary strategies and nanomedicine may open new avenues for preventing and managing bacteria‐associated cancers [380].

In moving forward, future studies could explore the gaps in our understanding of specific bacterial strains and their interactions with host cells in the context of cancer development. Additionally, investigating optimal combinations of conventional therapies and dietary interventions containing phytochemicals may yield insights into more effective cancer prevention strategies. Further research into the molecular mechanisms underlying the protective effects of natural‐based foods against bacterial infection‐induced carcinogenesis could help develop targeted preventive measures and therapeutic interventions.

## Author Contributions

**Md Sohel:** conceptualization; writing – original draft; writing – review and editing. **Suraiya Aktar:** writing – original draft. **Sanzida Khatun:** writing – original draft. **Sherif Hamidu:** writing – original draft. **Nishat Ulfat Nity:** writing – original draft. **Sandeep Kumar:** writing – original draft. **Md Sakhawat Hossain:** writing – original draft. **Md. Rifat Sarker:** writing – original draft. **Snygdha Rani Das:** writing – original draft. **Badhan Rani Dey:** writing – original draft. **Ali Mohamod Wasaf Hasan:** writing – original draft. **Khairul Islam:** writing – original draft. **Farhadul Islam:** review and editing. **Abdullah Al Mamun:** writing – original draft; writing – review & editing; supervision.

## Funding

No funding was received for this manuscript.

## Ethics Statement

The authors have nothing to report.

## Conflicts of Interest

The authors declare no conflicts of interest.

## Data Availability

Data sharing not applicable to this article as no datasets were generated or analyzed during the current study.
